# The interplay of carbon and nitrogen cycling driven by watershed microorganisms

**DOI:** 10.3389/fmicb.2025.1696238

**Published:** 2026-01-20

**Authors:** Guijia Sun, Qiang Zou, Bing Wang

**Affiliations:** 1 School of Environment and Resources, Southwest University of Science and Technology, Mianyang, China; 2 State Key Laboratory of Mountain Hazards and Engineering Resilience, Institute of Mountain Hazards and Environment (IMHE), Chinese Academy of Sciences (CAS), Sichuan, Chengdu, China; 3 China-Pakistan Joint Research Center on Earth Sciences, CAS-HEC, Islamabad, Pakistan

**Keywords:** carbon cycle, microbiome, microorganisms, nitrogen cycle, resistance gene, watershed ecology

## Abstract

Microorganisms play central roles in regulating carbon and nitrogen cycling across watersheds, driving processes such as organic matter decomposition, primary production, nitrification, and denitrification. Rapid advances in high-throughput sequencing and environmental monitoring have enabled unprecedented insights into the taxonomic diversity and functional capacities of microbial communities under global change. In this review, we synthesize findings from studies published in recent years to evaluate how hydrological connectivity, redox gradients, temperature shifts, and nutrient loading shape microbial metabolism across rivers, lakes, wetlands, and coastal interfaces. We further summarize emerging evidence on how antibiotic resistance genes (ARGs) propagate through these ecosystems and influence microbial functions. The integration of multi-omics technologies including metagenomics, metatranscriptomics, combined with ecological and biogeochemical modeling provides new opportunities to quantify microbe-mediated carbon sequestration and nitrogen transformation. Finally, we discuss current knowledge gaps, including the limited understanding of ARG-driven community restructuring and the insufficient mechanistic resolution of microbe–environment interactions under future climate scenarios. This review highlights the need for cross-scale, data-integrated frameworks to better predict how microbial processes regulate watershed-level biogeochemical cycles in a rapidly changing world.

## 1 Introduction

**Figure 1:**
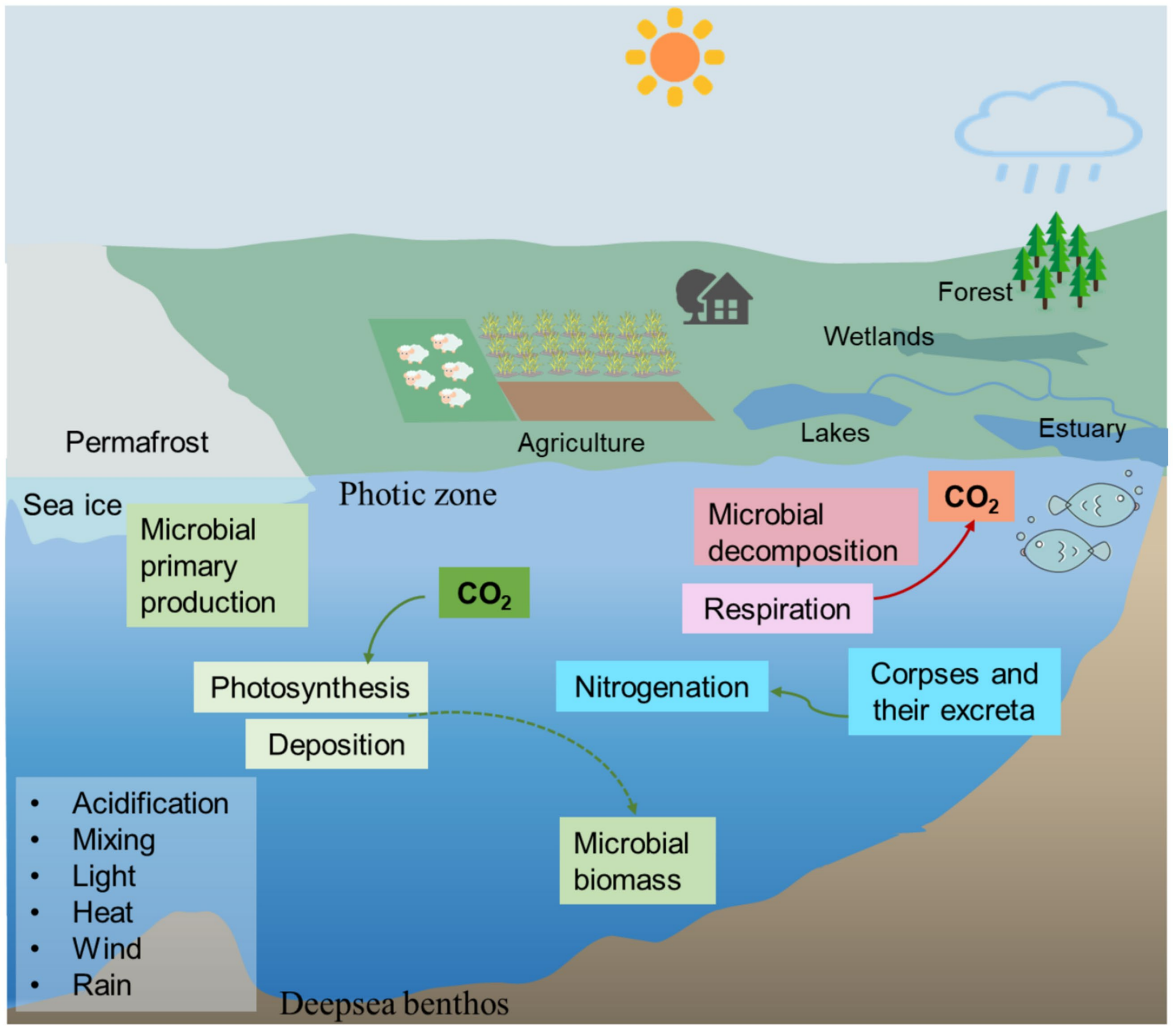
The role of watershed microorganisms.

Rivers and lakes are fundamental parts of the hydrological cycle and crucial sources of fresh water. They supply essential resources for human industrial and agricultural activities and support the functioning of ecosystems (Yang et al., 2021). Nevertheless, these aquatic ecosystems have been significantly degraded due to increasing disturbances from human activities and global environmental changes (Häder et al., 2020). In this context, microorganisms, which form the functional foundation of aquatic ecosystems, play an indispensable and central role in maintaining their stability and functionality (Figure 1) (Graham et al., 2016). As of May 19, 2025, the entries regarding the water metagenomic SRA (Sequence Read Archive) records in the NCBI (National Center for Biotechnology Information) database include marine metagenome (153,536), freshwater metagenome (103,949), wastewater metagenome (68,404), seawater metagenome (57,458), and aquatic metagenome (46276), which occupies a large proportion of the metagenomic data, indicated the vital role of microorganisms in watershed. Although numerous reviews have summarized microbial contributions to carbon and nitrogen cycling (Li P. et al., 2022; Battin et al., 2023; Wang Z. et al., 2023), most earlier works primarily focused on individual habitats (e.g., rivers or wetlands), single biogeochemical pathways, or data generated before 2022.

Recent advances including high-resolution metagenomics, genome-resolved microbial ecology, long-term watershed monitoring, and predictive biogeochemical modeling (Torres-Alvarado et al., 2019; Yang et al., 2022; Bai et al., 2024; Wang T. et al., 2024; Lu Y. M. et al., 2025) have generated substantial new knowledge that has not been integrated into previous reviews. In addition, microorganisms play a central role in mediating element export in aquatic ecosystems (Duhamel, 2025). Microbial mineralization processes drive the release and transport of dissolved organic and inorganic carbon, while fluvially transported particulate organic carbon reflects microbial processing of organic matter (Shi et al., 2024). Moreover, the accelerating spread of antibiotic resistance genes (ARGs) in natural ecosystems introduces additional ecological pressures that may alter microbial community functions (Larsson and Flach, 2022; Gao et al., 2024; Deng et al., 2025); however, this topic has rarely been reviewed together with watershed carbon and nitrogen cycling.

In this review, we synthesize the latest multi-omics discoveries across rivers, lakes, wetlands, estuaries, and coastal zones to provide a cross-system perspective on microbial processes. We highlight mechanistic insights from primary literature regarding redox-driven metabolism, hydrological connectivity, nutrient fluxes, temperature regulation, and environmental stressors. In addition, we discuss how ARG proliferation intersects with carbon and nitrogen transformations, an aspect overlooked in previous reviews. Finally, we outline emerging modeling frameworks and research gaps to guide future studies on microbe-mediated watershed biogeochemistry under global environmental change.

## 2 Watershed microbial composition and main function

### 2.1 Watershed microbial composition

Bacteria are omnipresent life forms, colonizing virtually every corner of the Earth, from the most extreme environments to the most hospitable ones. Within aquatic ecosystems, they form highly concentrated communities that exhibit a wide range of morphological, physiological, and ecological characteristics. The dominant phyla including Actinobacteria, Cyanobacteria, Acidobacteria, Caldeserica, Calditrichaeota, Verrucomicrobia, Chlorobi, Planctomycetes, Nitrospirae, Chloroflexi, Bacteriodetes, Firmicutes, and Proteobacteria were found in the freshwater ecosystems (Yadav et al., 2019). Proteobacteria, Bacteroidota, and Actinobacteriota were found dominated in Ocean (Table 1) (Kraemer et al., 2020; Acinas et al., 2021; Wang X. et al., 2024).

**Table 1:** Recent studies (2023–2025) on watershed microbial composition and functional genes.

Location	Sample source	Country	Sequence method	Key findings (dominant taxa/functional genes)	References
Rivers					
North American Rivers	River	United States/Canada	Metagenomics, Metatranscriptomics	Planktophila, Limnohabitans, Polynucleobacter; High abundance of vancomycin resistance genes (van)	Borton et al. (2025)
Himalayan Brahmaputra River	River Sediment	India	Metagenomics	Dominated by Pseudomonadota, Actinobacteria; Detection of ARGs: *aac(6′)-ly, TEM-116*	Sharma et al. (2024)
Yangtze River Source	River	China	16S rRNA Amplicon	Proteobacteria, Bacteroidota, Patescibacteria	Liu et al. (2025a)
Lakes					
Qinghai-Tibet Plateau Lakes	Lake Water	China	Metagenomics	Actinobacteriota, γ-Proteobacteria, Cyanobacteria; Functional genes for high-altitude adaptation	Feng et al. (2024)
Winam Gulf, Lake Victoria	Lake Water	Kenyan	Metagenomics	Actinobacteria, Cyanobacteria, Proteobacteria; Cyanotoxin biosynthesis genes	Hart et al. (2025)
Kenyan Lakes (Naivasha)	Lake Water	Kenyan	16S rDNA & Metagenomics	Picoplankton (Cyanobium, Synechococcus) dominate; ~77% of phn genes (phosphonate utilization) found in cyanobacteria	Zepernick et al. (2024)
High-altitude Saline Lakes	Lake Water	China	Metagenomics	Alphaproteobacteria, Rhodobacterales; Nitrogen cycle genes: nirB, nifH, nasA	Zhao et al. (2024)
Salt Lake	Lake Water	China	16S rRNA	*Proteobacteria*, *Bacteroidota*, and *Desulfobacterota*	Salmaso et al. (2024)
Ocean/coastal					
Northeastern Indian Ocean	Ocean Water	India	Metagenomics	Proteobacteria, Bacteroidota, Actinobacteriota; Depth-dependent community structure	Wang X. et al. (2024)
Arctic Ocean	Ocean Water	Arctic	Metagenomics	Colwellia, Luteolibacter, Flavobacterium; Community shifts driven by Atlantic water influx and sea-ice cover	Priest et al. (2023)
North and South Pacific and Atlantic Oceans, the Indian Ocean, and the South Australian Bight	Ocean Water		Metagenomics	*Salipiger, Novosphingobium, MarineAlpha9-Bin7, Sulfurimonas, Ahrensia, Thalassococcus*	Acinas et al. (2021)
Swan Lake, Weihai	Seagrass Sediment	China	Metagenomics	Woeseia, Zavarzinella; Diverse ARGs (sul1, mexW, bacA) and metal resistance genes	Cui K. et al. (2024)
Wetlands/others					
Calakmul Biosphere Reserve	Wetland Soil	Mexico	16S rRNA and Shotgun	Nocardioides, Haliangium, Gaiella; Functional diversity differences between conserved and urbanized zones	García-Estrada et al. (2024)
Global	Marine and Terrestrial	Global	Metagenomic DNA	Nitrospinota, Cloacimonadota, Planctomycetota; Clear marine–terrestrial divide in microbiome composition	Ruff et al. (2024)

### 2.2 Degradation of organic carbon compounds by bacteria and fungi

Microbial carbon conversion is a core process in the ecosystem carbon cycle, driving the interconversion of organic and inorganic carbon through decomposition, synthesis, and metabolism. This process is essential for linking carbon sources and sinks. Bacteria employ a variety of degradation pathways to eliminate aromatic organic pollutants, including polychlorinated biphenyls (PCBs), polycyclic aromatic hydrocarbons (PAHs), and pesticides. For example, strains like Achromobacter, Acinetobacter, and *Rhodococcus* spp. are known for their ability to metabolize PCBs (Taguchi et al., 2004; Dudášová et al., 2014; Liang et al., 2014; Pham et al., 2015; Shumkova et al., 2015; Atago et al., 2016; Xu et al., 2016; Blanco-Moreno et al., 2017; Horváthová et al., 2018; Wang et al., 2018). The degradation process typically involves initial oxidation by enzymes like biphenyl dioxygenase, followed by further breakdown into simpler compounds (Suman et al., 2021; Zubrova et al., 2021; Sciarrillo et al., 2022). Bacteria also metabolize small molecules via aerobic respiration or anaerobic fermentation, producing carbon dioxide or methane (Kumari and Das, 2023).

Fungi, particularly white-rot fungi, are crucial decomposers of plant biomass, especially lignin, a major component of lignocellulose (Floudas et al., 2012; Pawłowska et al., 2019; Lankiewicz et al., 2023). They release cellulose and hemicellulose through nonspecific oxidation, which can then be further degraded by bacteria. Fungi and bacteria often work synergistically: fungal metabolic products support bacterial growth, while bacterial decomposition releases carbon dioxide that can enhance fungal photosynthetic activities (Floudas et al., 2012).

The degradation efficiency of bacteria and fungi is influenced by environmental factors such as temperature, humidity, and microbial community composition. For example, optimal growth conditions for different strains can vary significantly (Ravi et al., 2022). Thermophilic fungi, for instance, can function effectively at higher temperatures and in partially anaerobic conditions, enhancing degradation rates (Ingersoll, 2023). In humid environments, microbial activity and enzyme diffusion are promoted, while dry or flooded conditions can inhibit decomposition or shift it to anaerobic pathways, potentially leading to methane production (Floudas et al., 2012). Competition for resources among microorganisms can also affect degradation rates, with rapid-growing bacteria potentially limiting fungal activity (Floudas et al., 2012).

### 2.3 Methanogenesis and methane oxidation

Methanogenesis and methane oxidation are opposing microbial processes that play significant roles in the carbon cycle. Methanogens, which are strict anaerobes, convert simple organic compounds such as carbon dioxide and acetic acid into methane under anaerobic conditions (Lyu et al., 2018). This process is influenced by factors such as temperature, salinity, substrate availability, pH, and the presence of compatible solutes in saline environments (Bueno de Mesquita et al., 2023). A recent study found that as climate change leads to alterations in lake temperature, salinity, and nutrient inputs, CH₄ emissions from lakes may become increasingly sensitive to environmental shifts (Cai et al., 2026). Methanogenesis contributes to the greenhouse effect but is balanced by methane oxidation.

Methane-oxidizing bacteria, primarily found in oxic zones, convert methane into carbon dioxide and water, significantly reducing methane emissions and mitigating the greenhouse effect (Duan et al., 2022; Zhang S. et al., 2022; Lenstra et al., 2023; Schmitz et al., 2023). Their activity is regulated by oxygen availability and temperature. Optimizing environmental conditions or enhancing microbial activity through biotechnological means can improve methane reduction efficiency in the future (Schmitz et al., 2023).

### 2.4 Carbon sequestration

Carbon sequestration is a critical process in the carbon cycle, involving the long-term storage of carbon in geological structures or organic matter. Microbial activities, such as the decomposition of plant residues and the formation of stable organic compounds like humus, contribute to carbon sequestration (Adamczyk et al., 2020; Sasse, 2023; Stukel et al., 2023). For example, white-rot fungi degrade lignin into phenolic compounds that combine with proteins and polysaccharides to form stable humus (Floudas et al., 2012). A recent study conceptualize soil organic matter stabilization through the “microbial carbon pump” (MCP), which integrates microbial catabolism and anabolism into two pathways—*ex vivo* modification and *in vivo* turnover—to explain how microorganism-driven processes lead to long-term carbon entombment in soils, highlighting the need for mechanistic research on soil carbon dynamics under global change (Liang et al., 2017). Other mechanisms involve the formation of complexes between root-derived tannins and fungal residues, which enhances carbon storage in ecosystems rich in mycorrhizal fungi (Adamczyk et al., 2020). These processes highlight the role of specific microorganisms in converting labile carbon into stable forms and maximizing soil and sediment carbon sequestration capacity.

Microbial management practices offer significant potential for enhancing carbon sequestration. For example, inoculating deep soil layers with specific microorganisms or adding reagents to inhibit bacterial carbon decomposition can promote fungal-dominated slow carbon cycling (Adamczyk et al., 2020; Sasse, 2023; Stukel et al., 2023). These approaches, combined with traditional agricultural practices and biotechnological innovations, can optimize microbial community structures and metabolic pathways, thereby improving carbon sequestration efficiency and contributing to carbon neutrality and sustainable agricultural development.

**Figure 2:**
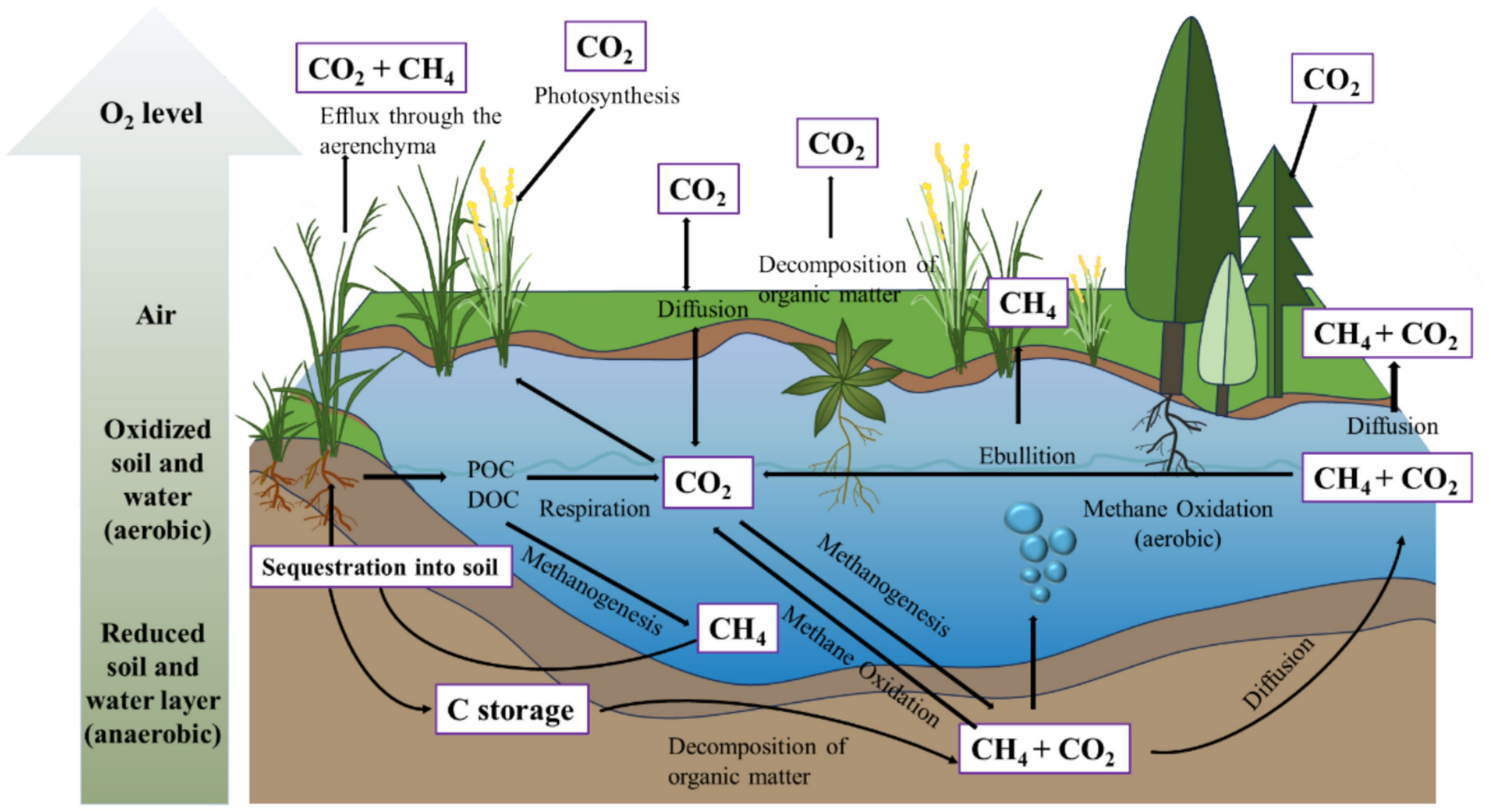
Representation of the water carbon cycle. The water carbon cycle involves multiple processes. Carbon sequestration is mainly propelled by photosynthesis, resulting in the accumulation of organic carbon in the forms of particulate organic carbon (POC) and dissolved organic carbon (DOC). Conversely, CO_2_ emissions arise through several pathways: respiration during the decomposition of organic matter, oxidation of methane (CH_4_), diffusion, and release of greenhouse gasses via plant aerenchyma tissues. Methane (CH_4_) emissions mainly originate from methanogenesis and subsequent release through plant aerenchyma, ebullition, and diffusion. When wetland ecosystems remain undisturbed, sequestered carbon can be stored within the soil profile for centuries (Bernal and Mitsch, 2012).

Microbial activities are central to the carbon cycle, linking carbon sources and sinks through degradation, methanogenesis, methane oxidation, and carbon sequestration (Figure 2). By regulating microbial processes and optimizing environmental conditions, we can enhance carbon sequestration and mitigate the greenhouse effect, ultimately promoting a more sustainable carbon cycle.

## 3 The nitrogen cycle in watersheds

### 3.1 Key components of the nitrogen cycle

Over the course of 3 billion years, the nitrogen cycle on earth has developed through biogeochemical and microbial processes that are interconnected via natural feedback mechanisms to maintain the oceanic nitrogen cycle in approximate equilibrium (Canfield et al., 2010). However, numerous watersheds globally are subjected to significant inputs of reactive nitrogen from human - related sources (Billen et al., 2013). The nitrogen cycle consists of several key components, including nitrogen fixation, nitrification, denitrification, and ammonification. These processes are detailed below. A recent study discovered that the presence of genes encoding *napAB/narGH* and the subsequent DNRA (Dissimilatory Nitrate Reduction to Ammonium) pathway in the majority of their metagenome-assembled genomes (MAGs) indicates that nitrate could potentially act as an alternative electron acceptor for *Deltaproteobacteria* (Hou et al., 2020).

**Figure 3:**
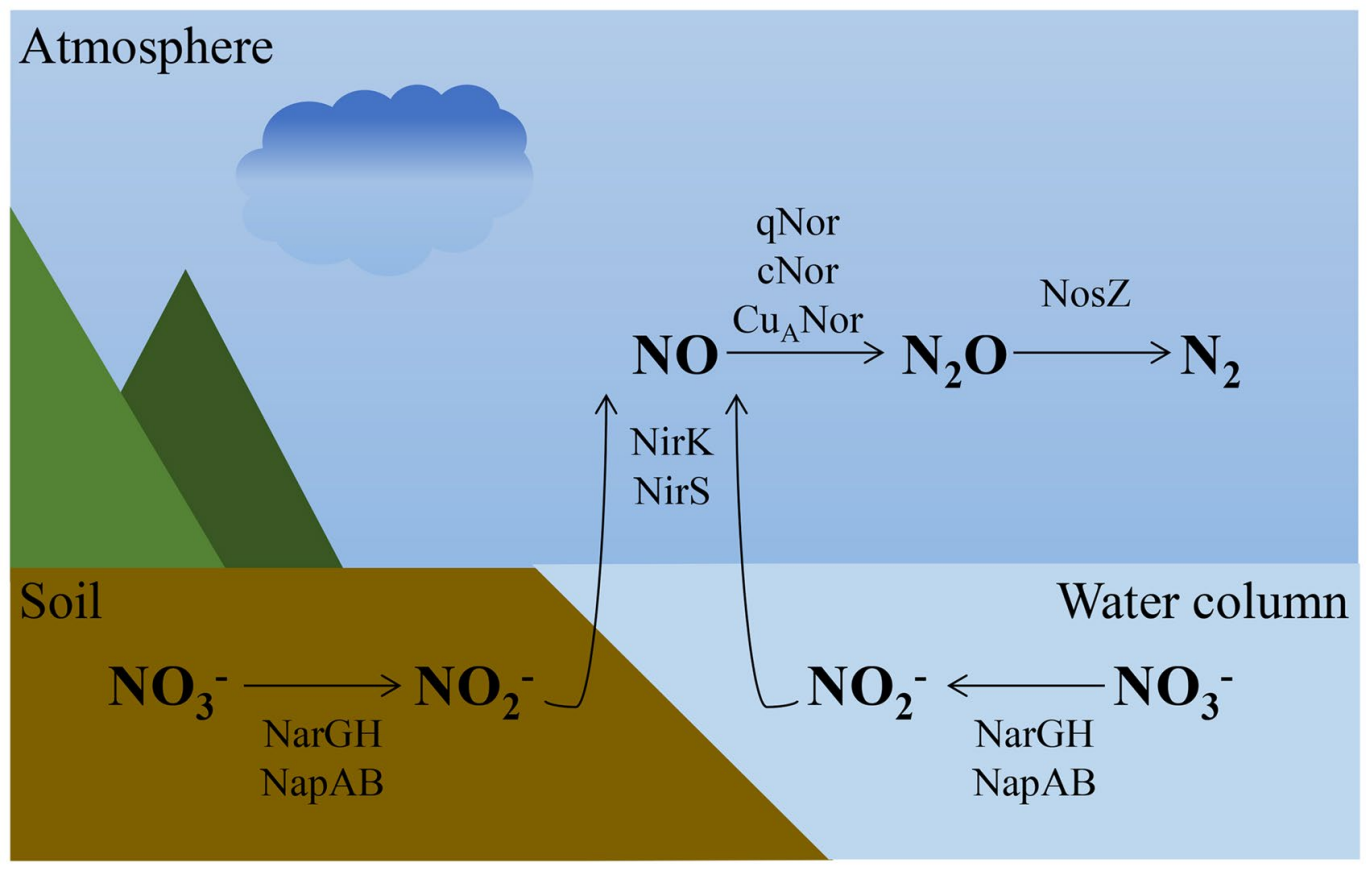
Representation of the water nitrogen cycle. Denitrification pathway in soil and water.

Despite the abundance of nitrogen in the atmosphere, it is typically the most limiting nutrient for plants because they are unable to directly utilize atmospheric nitrogen gas (N₂) (Figure 3). Plant growth relies entirely on fixed nitrogen, which is mostly obtained from the rhizosphere, the zone of soil surrounding and influenced by plant roots (Xu and Wang, 2023). Some diazotrophic prokaryotes can convert N₂ into ammonia (NH₃) through a process called biological nitrogen fixation (BNF), which maintains the nitrogen pool in the ecosystem (Pankievicz et al., 2019; Xu and Wang, 2023). Recent studies underscore the critical role of non-fixation nitrogen sources in sustaining coastal primary productivity. Quantitative assessments using stable isotope tracers and source-specific biomarkers have demonstrated that in many coastal ecosystems, riverine inputs, sewage effluents, and submarine groundwater discharge collectively contribute a substantial fraction—often exceeding 50%—of bioavailable nitrogen to vegetated habitats (e.g., seagrass beds and mangroves) (Pennino et al., 2014; Santos et al., 2021). The enzyme complex nitrogenase, which catalyzes nitrogen fixation, is highly conserved among diazotrophs and is inhibited by oxygen (Pankievicz et al., 2019; Xu and Wang, 2023). Nitrification is a key process in the nitrogen cycle, involving the sequential aerobic oxidation of ammonia (NH₃) to nitrate (NO₃^−^) via nitrite (NO₂^−^)(Koch et al., 2019). Nitrification ensures the conversion of ammonia, derived from the decomposition and mineralization of organic nitrogen in biomass, into a more soluble and oxidized form of nitrate. This process provides the substrate for denitrification, which returns nitrogen to the atmosphere (Schleper and Nicol, 2010). Denitrification is a vital process in the nitrogen cycle, in which nitrate (NO₃^−^) is progressively reduced to nitrite (NO₂^−^), nitric oxide (NO), nitrous oxide (N₂O), and ultimately dinitrogen (N₂) via a series of oxidoreductase enzymes (Torregrosa-Crespo et al., 2023). Denitrification is a natural filtering process that converts biologically usable nitrogen back into gaseous nitrogen, protecting water quality in rivers, soils, riparian wetlands, and river-bottom sediments (Billen et al., 2013; Ayvazian et al., 2021).

Ammonification: this process involves the decomposition of nitrogen-containing organic compounds by microorganisms, releasing ammonia (NH₃) or ammonium ions (NH₄^+^). Ammonification is a key step in converting organic nitrogen into inorganic forms that can be utilized by plants and other organisms (Li D. et al., 2022).

Throughout the nitrogen cycle, nitrogen undergoes transformations into various forms. Biological nitrogen fixation (BNF) refers to the process where microorganisms transform atmospheric nitrogen (N₂) into a form that is usable by living organisms (Liu et al., 2023). Nitrification is carried out by three groups of microorganisms: (1) ammonia oxidizers, which convert ammonia to nitrite; (2) nitrite oxidizers, which convert nitrite to nitrate; and (3) complete ammonia oxidizers, which directly convert ammonia to nitrate (Stein and Klotz, 2016). Denitrification refers to the anaerobic respiration process in which nitrite (NO₂^−^), nitric oxide (NO), and nitrous oxide (N₂O) are reduced to dinitrogen (N₂) (Stein and Klotz, 2016).

### 3.2 Microbial mediation of nitrogen processes

#### 3.2.1 Nitrogen fixation

A diverse range of nitrogen-fixing bacteria play significant roles in nitrogen assimilation in association with various plant groups, extending from microalgae to angiosperms. These bacteria include cyanobacteria, actinobacteria, and proteobacteria (Mathesius, 2022). The following sections focus on two key groups: cyanobacteria and actinobacteria.

Cyanobacteria are a diverse group of prokaryotes that perform oxygenic photosynthesis and can be found in marine, aquatic, and terrestrial environments worldwide. Some species of cyanobacteria can establish symbiotic associations with a wide range of plants. Symbiotic cyanobacteria typically belong to the filamentous genus Nostoc, although other symbiotic cyanobacteria, such as *Anabaena*, *Trichormus*, *Calothrix*, and *Chlorogloeopsis*, have also been identified (Rai et al., 2000; Adams and Duggan, 2008). Filamentous cyanobacteria can differentiate into various cell types, including akinetes (dormant spores), heterocysts (nitrogen-fixing cells), vegetative cells (carbon-fixing cells), and hormogonia (motile filaments) (Rai et al., 2000; Adams and Duggan, 2008). Hormogonia act as infective units during the establishment of symbiosis and exhibit short-distance dispersal under free-living conditions (Santi et al., 2013; Chang et al., 2019). Autotrophic cyanobacteria utilize energy from photosynthesis to drive nitrogen fixation, protecting their nitrogenase either physically (by isolating the nitrogenase in specialized heterocyst cells) or temporally (by fixing nitrogen only at night) (Pankievicz et al., 2019).

Actinorhizal plants are nodulated by the actinobacterium genus Frankia, named after the German biologist A. B. Frank (Chetri et al., 2022). These slow-growing, branched, filamentous Gram-positive soil actinobacteria are adapted to a wide variety of environments and have been found on all continents except Antarctica. Frankia produces three distinct cell types: filamentous vegetative hyphae, multicellular sporangia, and nitrogen-fixing vesicles (Benson and Silvester, 1993). Unlike rhizobia, free-living Frankia can fix nitrogen by forming specialized vesicles in the soil, which possess a multi-layered hopanoid-rich lipid envelope.

#### 3.2.2 Nitrification occurs in two distinct steps, each mediated by different microbial groups

The initial step of nitrification involves the oxidation of ammonia (NH₄^+^) to nitrite (NO₂^−^) by ammonia-oxidizing archaea (AOA) and ammonia-oxidizing bacteria (AOB). These microorganisms compete with heterotrophic bacteria and photosynthetic microorganisms for ammonium, which is their sole source of energy and nitrogen (French et al., 2021). The most common microorganisms involved in this reaction belong to the β-proteobacteria subclass of Proteobacteria (Niu et al., 2021). NH₄^+^ is sequentially oxidized to hydroxylamine (NH₂OH) and then to NO₂^−^. This process converts ammonia, which is derived from the decomposition and mineralization of organic nitrogen in biomass, into a more soluble and oxidized form of nitrate, providing a substrate for denitrification (Schleper and Nicol, 2010). The second step involves the oxidation of nitrite (NO₂^−^) to nitrate (NO₃^−^) by nitrite-oxidizing bacteria (NOB). These bacteria utilize nitrite as their sole energy source and convert it to nitrate through the enzyme nitrite oxidoreductase (NXR), which is part of the type II dimethyl sulfoxide reductase family of molybdoenzymes (Daims et al., 2016; Park et al., 2020).

#### 3.2.3 Denitrification

Denitrification is a vital process that safeguards river water quality by transforming nitrate (NO₃^−^) into nitrogen gas (N₂) in soils, riparian wetlands, and river-bottom sediments (Billen et al., 2013). Denitrifying bacteria drive this process, which sequentially reduces nitrate to nitrite (NO₂^−^), nitric oxide (NO), nitrous oxide (N₂O), and eventually dinitrogen (N₂) (Ayvazian et al., 2021). Many bacteria and archaea possess only partial denitrification capabilities. For instance, some ammonia-oxidizing chemolithotrophic bacteria can aerobically reduce NO₂^−^ to N₂O. Moreover, several eukaryotic organisms, including fungi and foraminifers, are capable of reducing NO₂^−^ or NO₃^−^ to N₂O or N₂, respectively. An unusual denitrifying bacterium, Candidatus *Methylomirabilis oxyfera*, consumes methane as an energy, reductant, and carbon source and reduces NO₂^−^ to N₂ by expressing a nitric oxide dismutase. This enzyme dismutates two NO molecules to O_2_ and N_2_ without producing the intermediate N_2_O (Stein and Klotz, 2016).

Several key factors influence the process of denitrification. Oxygen plays a crucial role, as changes in oxygen tension can trigger adaptive shifts between aerobic and anaerobic respiration in bacteria (Li et al., 2014). In oxygen-limited environments, nitrate serves as an alternative electron acceptor, allowing bacteria to continue generating energy through denitrification (Durand and Guillier, 2021). Moreover, the availability and type of carbon sources play a crucial role in determining denitrification efficiency. Carbon sources that have simpler metabolic pathways facilitate more efficient electron generation and transfer, thereby stimulating microbial growth and enhancing the completeness of denitrification (Wei et al., 2022). Understanding these factors is essential for optimizing carbon source selection, avoiding secondary pollution, and improving overall denitrification efficiency. In this context, we summarizes the key functional genes involved in microbial carbon and nitrogen cycling, including their marker genes, protein functions, and representative microbial groups (Table 2). These genes regulate major processes such as CO₂ fixation, methanogenesis, methane oxidation, nitrogen fixation, nitrification, denitrification, DNRA, and anammox. They serve as essential molecular markers for assessing microbial contributions to global biogeochemical cycles.

**Table 2:** Key functional genes in microbial carbon and nitrogen cycling.

Cycle	Process	Marker gene(s)	Protein/function	Dominant microorganisms	Representative references
Carbon	Calvin–Benson–Bassham (CBB) pathway	cbbL/cbbS, cbbM, prk	Rubisco + PRK for CO₂ fixation	Cyanobacteria; Proteobacteria; some Archaea	Thomas (2016), Balch et al. (2018), Smorti (2019)
Carbon	Methanogenesis	mcrA	Methyl-coenzyme M reductase, methanogenesis	Methanogenic Archaea (Methanosaeta, Methanosarcina)	Pikuta et al. (2000), Lau et al. (2020), Syrmou et al. (2022)
Carbon	Aerobic methane oxidation	pmoA, mmoX	Methane monooxygenases (CH₄ → CH₃OH)	Methanotrophic Proteobacteria (Type I and II)	Krause et al. (1997), Ikeda and Miyazaki (2011), Han and Han (2019), Liu et al. (2019)
Nitrogen	Biological N₂ fixation	nifH (± nifD/nifK, vnf/anf)	Nitrogenase, N₂ → NH₃	Cyanobacteria; heterotrophic bacteria; Archaea	Zhang and Xu (2006), Hand (2010), Sowmya (2018)
Nitrogen	Ammonia oxidation (AOB/AOA)	amoA (± amoB, amoC)	Ammonia monooxygenase, NH₃ → NH₂OH	AOB (Nitrosomonas, Nitrosospira), AOA (Nitrosopumilus, Nitrososphaera)	Ye et al. (2021), Zhang X. et al. (2022)
Nitrogen	Complete ammonia oxidation (comammox)	amoA (comammox clades A/B), nxrA/nxrB	Complete NH₃ → NO₃^−^ oxidation in single organism	Nitrospira (e.g., *N. inopinata*, *Ca.* N. kreftii)	Vayssier-Taussat et al. (2015), Johnson et al. (2018), Eisenstein (2019)
Nitrogen	Nitrite oxidation	nxrA, nxrB	Nitrite oxidoreductase, NO₂^−^ → NO₃^−^	Nitrospira, Nitrospina, Nitrobacter	Pang et al. (2012), Topor and Budson (2019)
Nitrogen	Dissimilatory nitrate reduction (nar/nap)	narG, napA	Nitrate reductases, NO₃^−^ → NO₂^−^	Proteobacteria in aquatic systems	Skinner et al. (2009), Vlassopoulou et al. (2016)
Nitrogen	Denitrification	nirK/nirS, norB, nosZ (clade I/II)	Stepwise NO₂^−^ → NO→N₂O → N₂	Proteobacteria (Marinobacter, Pseudomonas, Paracoccus, etc.)	Easom et al. (1998), Au et al. (2014), Kreibich et al. (2015)
Nitrogen	DNRA	nrfA	Cytochrome c nitrite reductase, NO₂^−^ → NH₄^+^	Facultative anaerobes (Shewanella, Geobacter, etc.)	Hacker and Barlow (2015), Wahiduzzaman et al. (2017)
Nitrogen	Anammox	hzsA, hzo	Hydrazine synthase/oxidoreductase, NH₄^+^+NO₂^−^ → N₂	Planctomycetes (*Ca.* Scalindua, Brocadia, Kuenenia)	Kuechler et al. (2012), Pak et al. (2015), Clapham (2020)
Nitrogen	Assimilatory nitrate/nitrite reduction	nasA, nasB/nirA	Assimilation of nitrate/nitrite to biomass NH₄^+^	Cyanobacteria; many heterotrophic bacteria	Takeuchi et al. (2005), Hao et al. (2015), Mishra et al. (2017)

## 4 Microbial mediation of interactions between carbon and nitrogen cycles

### 4.1 Coupled processes

Microbial activities are instrumental in driving nitrogen mineralization and regulating the rate and direction of the nitrogen cycle. Within watershed ecosystems, microorganisms are pivotal in both the carbon and nitrogen cycles. Their actions shape the transformation processes of carbon and nitrogen, and through intricate interactions, they influence ecosystem functions and global biogeochemical cycles. In the carbon cycle, microorganisms decompose organic matter and respire, thereby releasing carbon dioxide (Gougoulias et al., 2014; Wu et al., 2024). Under anoxic conditions, the microbial decomposition of organic matter shifts from aerobic respiration (primarily releasing CO₂) to anaerobic respiration, where terminal electron acceptors such as sulfate, nitrate, or ferric iron are used (LaRowe and Van Cappellen, 2011; Regnier et al., 2013). This process leads to the predominant production of bicarbonate (HCO₃^−^) rather than carbon dioxide, which can influence carbon cycling in several key ways: (1) Bicarbonate ions are more soluble and less volatile than CO₂, enhancing the retention of dissolved inorganic carbon (DIC) in sediment porewater and overlying water columns; (2) In aquatic and marine sediments, bicarbonate can precipitate as carbonate minerals (e.g., CaCO₃) under suitable geochemical conditions, leading to long-term carbon burial; and (3) The generated bicarbonate may also be transported via groundwater or hydrological flow to the ocean, where it contributes to the marine alkalinity pump and oceanic carbon storage (Barber et al., 2017; Boudreau et al., 2018). Additionally, they enhance the photosynthetic carbon sequestration of aquatic plants and algae, impacting the formation of soil organic carbon and long-term carbon storage.

In the nitrogen cycle, microorganisms decompose organic substances such as plant residues and animal manure, converting organic nitrogen into inorganic nitrogen (e.g., ammonia and nitrate) through a process called mineralization (Hu et al., 2022). This process is a key step in the nitrogen cycle, providing absorbable nitrogen nutrients for plants and supporting microbial growth (Xie et al., 2022). Bacteria and fungi in water bodies can break down organic nitrogen in plant root secretions and litter, converting it into ammonia and increasing the content of available nitrogen in water bodies (Eisenhauer et al., 2017). Moreover, during nitrogen fixation, microorganisms transform atmospheric nitrogen gas into ammonia, thereby augmenting the available nitrogen within the ecosystem and propelling the nitrogen cycle (Kuypers et al., 2018). Microbial activities also regulate the nitrogen cycle’s rate and direction by influencing soil pH and redox conditions, thereby affecting nitrification and denitrification (Ren et al., 2024). Nitrification is the process of oxidizing ammonium nitrogen to nitrate nitrogen, primarily carried out by ammonia-oxidizing and nitrite-oxidizing bacteria (van Kessel et al., 2015). Denitrification, on the other hand, is the process of reducing nitrate nitrogen to nitrogen gas, typically performed by denitrifying bacteria under anaerobic conditions (Smriga et al., 2021). For example, microbial nitrification can oxidize ammonia to nitrate, or through denitrification, nitrate can be reduced to nitrogen gas or dinitrogen monoxide (N₂O) (Lu et al., 2014), thereby regulating the nitrogen cycle’s rate and direction.

The interactions between carbon and nitrogen processes can be synergistic or antagonistic. These interactions reflect the mutual promotion or restraint among different biological and abiotic factors in the carbon and nitrogen cycle processes. Synergistic effects occur when the progress of one process provides favorable conditions for another, enhancing the overall ecosystem functions, while antagonistic effects occur when one process inhibits the other. The synergistic effects in the carbon-nitrogen cycle are primarily manifested in nitrogen fixation and carbon fixation, as well as nitrification and carbon mineralization. Microorganisms carry out nitrogen fixation to convert atmospheric nitrogen gas into ammonia, providing nitrogen nutrition for plant growth and a nitrogen source for microbial growth, thereby promoting the decomposition and utilization of organic carbon by microorganisms (Xu and Wang, 2023). In ecosystems where nitrogen-fixing bacteria and plants coexist, nitrogen-fixing bacteria provide nitrogen to plants through nitrogen fixation, while plants supply organic carbon to the nitrogen-fixing bacteria through photosynthesis (Mus et al., 2016). This synergistic effect enhances the carbon and nitrogen fixation efficiency of the ecosystem (Li and Li, 2023). Additionally, aquatic microorganisms consume a large amount of oxygen through nitrification, oxidizing ammonia to nitrate (Liu and Wang, 2012; Stein, 2019; Wright and Lehtovirta-Morley, 2023). During this process, microorganisms obtain energy through ammonium oxidation and utilize organic carbon as an electron donor, promoting carbon mineralization (Yuan et al., 2025). This synergy helps maintain the carbon-nitrogen balance in water areas.

The antagonistic effects in the carbon-nitrogen cycle are mainly manifested in the competition between denitrification and carbon fixation, as well as the inhibition of carbon fixation by excessive nitrogen. In oxygen - deficient environments, microorganisms prefer to use organic carbon to carry out denitrification, reducing nitrate to nitrogen gas and releasing it into the atmosphere (Wang S. et al., 2025). While this process helps to remove nitrogen pollution from water bodies, it may also lead to the excessive consumption of carbon, thereby affecting the accumulation of organic carbon in aquatic environments (Zheng et al., 2025). Excessive nitrogen can inhibit microbial decomposition of organic carbon, resulting in decreased carbon fixation efficiency (Zheng et al., 2025). For example, in farmlands with excessive fertilization, excess nitrogen fertilizer can alter the structure of the microbial community, inhibiting nitrogen-sensitive microorganisms and affecting the decomposition and fixation of soil organic carbon (Wu M. et al., 2025).

### 4.2 Microbial community dynamics

#### 4.2.1 The impact of carbon and nitrogen availability changes on microbial community structure

Microorganisms influence carbon and nitrogen dynamics in ecosystems through processes like organic matter decomposition, photosynthetic carbon sequestration, nitrogen fixation, nitrification, and denitrification (Cui Y. et al., 2024; Wani et al., 2025). However, the rates of the carbon and nitrogen cycles are not solely driven and regulated by the microbial community; they also impact microbial community structure and function through feedback mechanisms (Lu N. et al., 2025).

The availability of carbon, which is crucial for microbial growth and metabolism, directly influences microbial activity and community structure. When carbon sources are sufficient, microbial biomass and metabolic activity typically increase, accelerating organic matter decomposition and carbon dioxide release (Yu et al., 2025; Zhang G. et al., 2025). However, the type and quality of carbon sources also influence microbial community composition (Dang et al., 2024; Yu et al., 2025). For instance, easily decomposable carbon sources (e.g., glucose) promote the proliferation of rapidly growing bacteria (e.g., Proteobacteria), while more complex carbon sources (e.g., lignin) favor fungi with intricate metabolic pathways (e.g., Ascomycota) (Yu et al., 2025). Additionally, carbon availability affects microbial carbon use efficiency (CUE), which is the proportion of absorbed carbon used for microbial growth (Dang et al., 2024).

Nitrogen is a key element for microbial protein and nucleic acid synthesis, significantly impacting microbial growth and metabolism. Nitrogen addition can substantially alter microbial community structure, particularly the proportions of fungi and bacteria (Li S. et al., 2025; Li X. et al., 2025). Studies have shown that nitrogen addition often inhibits fungal growth while promoting bacterial proliferation (Wang X. et al., 2023), likely because fungi have higher nitrogen requirements and more complex nitrogen metabolic pathways. Furthermore, nitrogen addition can influence the abundance and diversity of functional genes in microorganisms (Lladó et al., 2017; Wang and Kuzyakov, 2024; Zhang Y. et al., 2025). For instance, nitrogen addition can boost the abundance of functional genes related to the nitrogen cycle (such as those involved in ammonium oxidation and nitrification) while potentially decreasing the abundance of functional genes related to the carbon cycle (such as those involved in cellulose decomposition) (Ameer et al., 2025). These changes can further affect the dynamic balance of the carbon-nitrogen cycle, such as altering soil organic carbon formation and decomposition rates.

In addition, nutritional limitations or imbalances during the carbon-nitrogen cycle can alter microbial metabolic pathways and community structure. In environments with abundant carbon but limited nitrogen, microorganisms preferentially utilize the limited nitrogen for growth, inhibiting high-nitrogen-demanding microorganisms while favoring those that can efficiently use limited nitrogen (Reese et al., 2018; Bao et al., 2024; Han et al., 2025). Conversely, in environments with abundant nitrogen but limited carbon, microbial growth and metabolism may be restricted, inhibiting microorganisms that efficiently decompose organic carbon (Lian et al., 2024). Additionally, these imbalances can affect microbial functions. Excessive nitrogen may inhibit microbial decomposition of organic carbon, reducing carbon fixation efficiency, while limited carbon sources may lead microorganisms to decompose more organic nitrogen, affecting nitrogen cycle efficiency (Zhang et al., 2023; Lu F. et al., 2025; Zhou et al., 2025).

#### 4.2.2 The interaction between microbial community composition and biogeochemical cycles

The feedback mechanism between microbial community composition and biogeochemical cycles is a critical interaction within ecosystems. Microorganisms, being the primary drivers of biogeochemical cycles, directly influence the rates and directions of element cycling, including carbon, nitrogen, and phosphorus (Li Z. et al., 2025; Liu et al., 2025a). In turn, changes in biogeochemical cycles feedback to regulate microbial community structure and function by modifying environmental conditions such as nutrient availability, soil pH, and redox potential (Iqbal et al., 2025). This interplay creates a complex feedback system that is essential for ecosystem functioning and the global biogeochemical cycle.

In watershed ecosystems, microorganisms propel the cycles of carbon, nitrogen, and phosphorus via processes like organic matter decomposition, carbon dioxide fixation, and nitrogen transformation, thus impacting biogeochemical cycles (Liu et al., 2025b). In the carbon cycle, they break down complex organic carbon into simple inorganic carbon, release carbon dioxide, and provide a carbon source for photosynthesis in aquatic plants and algae, promoting organic carbon fixation (Wang X. et al., 2024). In the nitrogen cycle, microorganisms control the availability of nitrogen for plants through essential processes like nitrogen fixation, nitrification, and denitrification, thereby affecting nitrogen loss and water quality in the watershed. For instance, studies have found a positive correlation between microbial nitrogen metabolism and nitrogen concentration in nutrient-affected river and lake corridors (Chen et al., 2025). These processes not only determine the conversion rates of carbon and nitrogen in the ecosystem but also affect overall ecosystem function and stability.

## 5 Impact of environmental factors on microbial activity in watersheds

### 5.1 Physical and chemical factors

**Figure 4:**
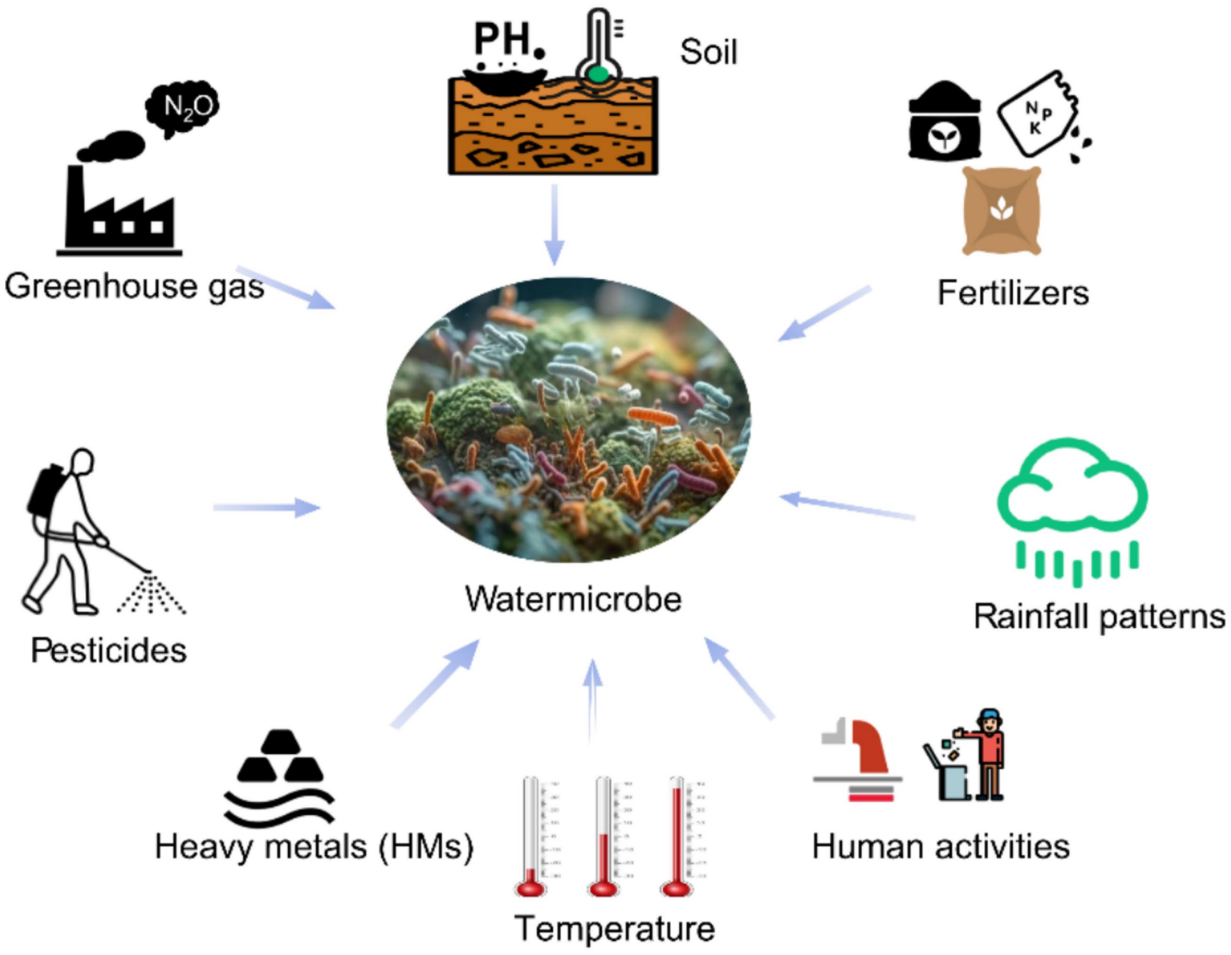
Impact of environmental factors on microbial activity in watersheds.

In watershed ecosystems, microbial activity is greatly affected by various physical and chemical factors such as temperature, moisture, fertilizers, rainfall, greenhouse gas emissions, pesticides, human activities, heavy metals and soil pH (Figure 4). These factors shape microbial community structure and function, thereby affecting key biogeochemical cycles such as carbon and nitrogen transformations.

Temperature is a critical driver of microbial metabolism, influencing enzyme activity and community composition. Global climate models forecast that the global average temperature will rise by 0.3–4.8 °C by the end of the 21st century (Intergovernmental Panel on Climate Change, 2014; Impa et al., 2019). While moderate temperature increases (e.g., 3 °C) can enhance microbial respiration (Nydahl et al., 2013), extreme temperatures have contrasting effects. Elevated temperatures (>40 °C) may inactivate most microbial enzymes, except for those in heat-resistant microorganisms (e.g., thermophilic bacteria), decreasing the decomposition rate of organic matter and resulting in carbon accumulation (Walker et al., 2018). Low temperatures (<10 °C) reduce microbial activity, limiting enzymatic reactions and slowing the decomposition of soil organic carbon (SOC). This explains why cold ecosystems (e.g., tundra, boreal forests) store large amounts of organic carbon that would rapidly decompose under warmer conditions (Davidson and Janssens, 2006). Microorganisms have developed various adaptation mechanisms to cope with temperature variations. To adapt to low temperatures, microorganisms increase the proportion of unsaturated fatty acids in their cell membranes to preserve membrane fluidity and functionality (Allison and Martiny, 2008). Under thermal stress, microbes upregulate heat-shock proteins (e.g., chaperonins like GroEL) to prevent protein denaturation and assist in refolding damaged proteins (Allison and Martiny, 2008). Rising temperatures directly affect microbial metabolism by altering enzyme kinetics and membrane fluidity, typically increasing soil respiration (Crowther et al., 2016). A statistically-based probabilistic forecast, derived from a Bayesian hierarchical model using country-level data, estimates a likely global temperature increase of 2.0–4.9 °C by 2100 (median 3.2 °C) and concludes that achieving under 1.5 °C warming would require a dramatically accelerated reduction in carbon intensity beyond recent trends (Nissan et al., 2023), which could significantly stimulate anaerobic processes such as methanogenesis and denitrification. Incorporating these habitat-specific warming trajectories into microbial-explicit models is essential for accurately predicting future carbon and nitrogen cycles (Yvon-Durocher et al., 2012).

pH affects microbial activity by influencing membrane stability, enzyme function, and community composition. Different microorganisms have varying pH tolerances. In acidic soils (pH < 5), fungal biomass and activity often dominate over bacteria due to their tolerance to low pH and ability to decompose recalcitrant compounds like lignin. Bacterial communities are typically less abundant and diverse under such conditions (Fierer and Jackson, 2006). Soil pH is a strong predictor of bacterial richness, diversity, and overall community composition (Figure 3) (Fierer and Jackson, 2006). Under neutral and alkaline conditions, bacteria dominate and promote the decomposition of easily degradable organic carbon. In the nitrogen cycle, nitrifying bacteria prefer neutral and alkaline environments. Ammonia-oxidizing bacteria (AOB) such as *Nitrosomonas* spp. exhibit optimal activity at neutral to alkaline pH (7–8), with a sharp decline in nitrification rates below pH 6 due to reduced ammonia (NH₃) availability and enzyme instability (Nicol et al., 2008). Extremely acidic or alkaline environments can inhibit the activity of nitrogen-fixing bacteria.

Moisture affects the diffusion of oxygen and substrate utilization, influencing the distribution of aerobic and anaerobic bacteria. Below −1.5 MPa water potential (≈30% water-holding capacity, WHC), microbial activity becomes diffusion-limited. Extracellular enzymes cannot migrate through thin water films, and cytoplasmic dehydration leads to dormancy in most taxa, except for xerotolerant Actinobacteria (Schimel et al., 2007). Under drought conditions, nitrification is inhibited, reducing the supply of nitrate. Conversely, moist or flooded conditions are highly favorable for denitrification. Under water-saturated conditions (>80% water-filled pore space), denitrification rates increase significantly due to oxygen limitation, forcing microbes to utilize nitrate (NO₃^−^) as an alternative electron acceptor. This effect is particularly pronounced when labile carbon (e.g., dissolved organic carbon) is abundant, providing energy for N₂O reductase activity (Leff et al., 2015). N₂O fluxes peak at intermediate moisture levels (60–80% WFPS) but decline under sustained flooding as carbon becomes limiting (Leff et al., 2015). For the carbon cycle, flooded conditions weaken the decomposition activity of aerobic bacteria, promoting anaerobic respiration and the production of organic acids. Moderately moist conditions are conducive to the progress of the carbon cycle.

Carbon, nitrogen, and phosphorus are key nutrients that directly affect microbial growth and function. The carbon cycle is primarily influenced by the carbon-nitrogen (C: N) ratio. High C: N ratios limit microbial activity due to nitrogen deficiency, reducing decomposition rates. In the nitrogen cycle, nitrogen-rich conditions inhibit nitrogen-fixing bacteria but promote the activity of nitrifying bacteria. Chronic nitrogen addition (>50 kg N ha^−1^ yr.^−1^) suppresses diazotrophs (e.g., Rhizobium spp.) by 40–60%, while stimulating ammonia-oxidizing bacteria (AOB) such as Nitrosomonas by 2–3 fold (Leff et al., 2015). Phosphorus limitation reduces microbial biomass and enzyme synthesis. Under phosphorus limitation (soil available *p* < 5 mg kg^−1^), microbial biomass C: P ratios exceed 50:1, forcing cells to allocate 30–50% of total carbon flux to phosphatase production, at the expense of growth-related enzymes (e.g., cellulases) (Leff et al., 2015).

In summary, temperature, pH, moisture, and nutrient availability jointly regulate microbial functions and activities, thereby influencing the carbon and nitrogen cycles in watersheds.

### 5.2 Human activities

Human activities significantly impact the structure and activity of microbial communities in watersheds by altering physical and chemical factors such as soil nutrients and temperature. Anthropogenic activities have doubled the amount of reactive nitrogen in the global nitrogen cycle (Fowler et al., 2013). N₂O, a potent greenhouse gas, primarily originates from soil microbial processes. The artificial input of nitrogen intensifies these microbial processes, including nitrification (Jung et al., 2014). Bacterial interactions significantly influence ecological functions in response to human-induced disturbances and changes in soil structure (Xiong et al., 2022).

Empirical studies have demonstrated that agricultural management practices and soil heterogeneity can substantially influence soil nutrient conditions, which in turn affect bacterial distribution and interactions (Bailey et al., 2013; Wan et al., 2021). The widespread use of chemical and organic fertilizers has become common in farming. However, long-term use of chemical fertilizers can lead to soil acidification, reduced microbial diversity, and inhibited activity. Chronic nitrogen addition (>50 kg N ha^−1^ yr.^−1^) reduces soil C: N ratios from 25–30 to 15–20 within a decade, shifting microbial metabolism from carbon limitation to nitrogen limitation. This nitrogen-induced shift decreases organic matter decomposition efficiency by 20–40%, as microbes downregulate nitrogen-acquiring enzymes (e.g., proteases) and allocate less carbon to growth-related functions under nitrogen saturation (Leff et al., 2015).

Human activities also introduce pollutants like heavy metals and pesticides, which pose severe threats to soil health and microbial activity. Heavy metal pollution, characterized by its insidious, long-term, and irreversible nature, leads to significant degradation of soil ecological structure and function (Chen et al., 2023). Pesticides can inhibit the activities of nitrogen-cycling microorganisms. Additionally, climate change, particularly increased temperatures, has both short-term and long-term effects on microbial communities. Short-term warming (days to weeks) increases microbial metabolic rates by 2–3 fold per 10 °C rise (Q₁₀ effect), driven by enhanced enzyme kinetics. This accelerates labile carbon mineralization, often doubling CO₂ fluxes from soils (Davidson and Janssens, 2006). In the long term, however, it reduces microbial diversity. After >5 years of warming, thermophiles (e.g., Thermus spp., Geobacillus) dominate, while mesophiles decline by 40–60%. This community shift reduces functional diversity and lowers decomposition efficiency of complex organics (e.g., lignin) (Walker et al., 2018).

Drought conditions, exacerbated by weakened rainfall patterns, inhibit nitrification and intensify carbon decomposition. Under drought stress (< −1.5 MPa), nitrification rates decline by 70–90% due to ammonia-oxidizing bacteria (AOB) sensitivity to low water potential, while fungi dominate decomposition via hyphal networks that access isolated water films, preferentially degrading lignin-rich substrates (Schimel et al., 2007). Despite these influences, microbial communities in watersheds exhibit adaptation and recovery potential. Functional redundancy among microbial taxa ensures ecosystem processes (e.g., cellulose degradation) persist under disturbance. For example, >20 bacterial genera encode cellulases, allowing decomposition to continue even if dominant taxa are suppressed (Allison and Martiny, 2008). Microbial communities can also switch to alternative metabolic pathways to maintain activity. Moreover, microorganisms can work together through mutualistic relationships to resist external environmental interference.

In summary, physical and chemical factors and human activities jointly shape the activities and functions of microbial communities in watersheds. Ongoing research continues to reveal the intricate relationships between environmental factors and microorganisms, highlighting the importance of understanding microbial adaptation mechanisms to achieve ecological balance.

## 6 The role of resistance genes in watershed microbial communities

### 6.1 Antibiotic resistance genes

With the continuous development of molecular biology and high-throughput sequencing technologies, our understanding of resistance genes (ARGs) in environmental microbial communities has deepened significantly. Watersheds, as important habitats for natural microbial communities and intersections for human activities (such as agriculture, urban sewage, and industrial emissions), have become significant “reservoirs” for the accumulation and spread of resistance genes (Wright, 2007; Serrana et al., 2025; Xiao et al., 2025; Yang et al., 2025). These resistance genes are not only present in pathogenic bacteria but are also widely distributed in the natural environment, forming the so-called “resistome.” This resistome has profound implications for ecosystem stability and public health (Wright, 2007). Antibiotic resistance genes are genetic elements in bacteria that encode proteins or mechanisms that resist the effects of antibiotics (Hernando-Amado et al., 2019). These genes include both intrinsic resistance genes that naturally exist in environmental microorganisms and acquired resistance genes that spread through selection pressure due to human activities (Martínez et al., 2007). The extensive use of antibiotics in various sectors, such as animal husbandry, medical treatment, and aquaculture, leads to the excretion of antibiotics and their metabolites through feces and their subsequent discharge into the environment via sewage treatment plants. These compounds ultimately enter rivers, lakes, and other water bodies (Larsson and Flach, 2022; Gao et al., 2024; Deng et al., 2025). Additionally, animal feces and fertilizers used in agricultural production often carry resistant bacteria, which are washed into watersheds during rainfall and irrigation (Rizzo et al., 2013; Arsand et al., 2020).

In watershed water bodies, commonly detected resistance genes include those that confer resistance to multiple classes of antibiotics, such as β-lactams, tetracyclines, sulfonamides, and fluoroquinolones (Singh et al., 2016; Sola et al., 2022; Malik et al., 2023; Yamaguchi et al., 2024) (Table 3). Class 1 integron *intI1* and *sul1* are the most consistently detected markers across continents; they co-vary with human fecal and wastewater signals and are widely used as environmental AMR indicators (Gillings et al., 2015). Tetracycline and β-lactam resistance are common: *tet* (A/M/W/X) and *bla* families (especially *blaTEM*, *blaCTX-M*) are frequently observed in rivers and lakes influenced by WWTPs; carbapenemases occur at low frequency but are detectable in highly impacted systems (Schachner-Groehs et al., 2024).

**Table 3:** Global ARGs in aquatic environments.

Continent	Common ARG classes/marker genes (frequent or notable)	Typical sources and settings	Representative waters (examples)	Key references
Asia	sulfonamides: sul1/sul2; tetracyclines: tetA/tetM; β-lactams/ESBLs: blaTEM, blaCTX-M; quinolones: qnr; class 1 integron: intI1; occasional carbapenemases (blaNDM, blaKPC) where impacted; co-selection with metals	Municipal and hospital wastewater, intensive aquaculture, pharmaceutical manufacturing effluents; large river corridors with dense population	Yangtze River system (China); industrially impacted QTP reservoirs; Indian lakes receiving pharma waste	Huang et al. (2019), Jiang et al. (2024)
Europe	intI1, sul1 ubiquitous; tetM/tetA, blaTEM common; ESBLs (blaCTX-M) seasonal/variable; low but detectable carbapenemase markers (blaOXA-48-like, blaKPC)	WWTP effluents and combined sewer overflows into rivers; coastal seas with urban inputs	Danube River (multi-country); Lahn River (Germany); English Channel and North Sea plankton	Herrig et al. (2020), Schachner-Groehs et al. (2024)
North America	intI1, sul1/sul2, tetW/tetX/tetB, erm(B); β-lactamases (blaTEM/CTX-M) sporadic; ARG load tracks human fecal signals	Municipal wastewater and septic systems; urbanized streams; Great Lakes near outfalls	US rivers/streams nationwide survey; septic-impacted coastal streams; Great Lakes near urban shorelines	Keely et al. (2022), Reddy and Kiledal (2024)
Africa	sul1/sul2, intI1 high in urban sediments; mixed tet genes; lakes with high human pressure show diverse multidrug ARGs	Rapid urbanization with limited sanitation; direct discharge to wetlands/lakes; fisheries	Urban wetlands (Nigeria); Lake Victoria (Kenya)	Adelowo et al. (2018)
South America	sul1, intI1, tet genes frequently reported; ESBLs (blaTEM/CTX-M) near urban WWTPs; qnr markers in impacted basins	Urban rivers receiving WWTP effluents; agricultural runoff; informal settlements	Reports across urban rivers (Brazil/Andean basins) with indicator genes (proxy literature generalizes patterns)	Kusi et al. (2022)
Oceania	Indicator set intI1, sul1/sul2, tet genes around WWTP outfalls; aquaculture-linked ARGs where intensive operations occur	Municipal wastewater and aquaculture sites; coastal receiving waters	Australian urban rivers/coasts; aquaculture facilities in the region (SEA-focused evidence extrapolates patterns)	Siri et al. (2023)
Antarctica	Very low background; human-impacted stations can show intI1/sul1 at trace levels (evidence sparse and site-specific)	Research station wastewater footprints	Localized near outfalls; not widespread	Gillings et al. (2015)

These genes primarily exist as plasmids, integrons, or transposons and can be disseminated among different bacterial species via horizontal gene transfer (HGT). This process enables originally susceptible bacteria in the environment to acquire resistance (Su et al., 2014). Research has indicated that even under the selective pressure of low concentrations of antibiotics, horizontal gene transfer (HGT) can efficiently facilitate the spread of resistance genes, thereby establishing a stable and extensive environmental reservoir of resistance genes (Munita Jose and Arias Cesar, 2016; Ju et al., 2019). Future research should integrate multi-omics (Su et al., 2021a; Su et al., 2021b; Wang T. et al., 2025) approaches with field observations to enhance our understanding of microbial roles in key biogeochemical cycles.

### 6.2 Implications for ecosystem health

Recent studies have demonstrated that the spread of antibiotic resistance genes is not only an indicator of anthropogenic pollution but also a factor capable of restructuring microbial community metabolism. ARG acquisition can modify cellular energy allocation, alter stress-response pathways, and influence the abundance of key functional guilds involved in C and N cycling. For example, exposure to antibiotic residues or ARG-rich microbial consortia has been shown to suppress microbial respiration and shift carbon degradation pathways (Wu Z. et al., 2025). Similarly, Soil organic carbon inputs can modulate key genes involved in the pyruvate/acetyl-CoA metabolic pathway, including ackA and pta, thereby shifting microbial communities from merely stress-tolerant to highly resistant phenotypes and fostering the accumulation of ARGs (Zhang et al., 2024).

The presence and spread of resistance genes pose potential threats to human health and alter the microbial structure and function of entire ecosystems (Thornber et al., 2020; Mettenleiter et al., 2023). First, the accumulation of resistance genes changes the competitive landscape of environmental microbial communities. When high concentrations of antibiotics or other chemical pollutants (such as heavy metals and pesticides) are present in watersheds, bacteria with resistance genes have a survival advantage and reproduce preferentially. This can result in imbalances in community structure, which in turn can impact ecological functions such as nutrient cycling, decomposition, and energy flow in water bodies (Kraemer et al., 2019). Second, resistance genes can rapidly spread between different bacteria in the environment through horizontal gene transfer, causing some pathogens to acquire multiple resistances. This increases the difficulty of clinical treatment. For example, common pathogens in watersheds, such as *Escherichia coli*, *Salmonella*, and *Staphylococcus aureus*, often exhibit resistance to multiple antibiotics after acquiring resistance genes. This greatly reduces the effectiveness of post-infection treatments and may even lead to the emergence of “super bacteria” (Barber, 1961; Poeta et al., 2008). Moreover, the enrichment of resistance genes in the environment may have direct or indirect toxic effects on aquatic organisms (Barlow and Hall, 2003). Aquatic organisms exposed to low-dose antibiotics over long periods may experience physiological, behavioral, and reproductive impairments. For instance, some studies have found that antibiotic pollution can interfere with the endocrine systems of fish and invertebrates and may lead to declines in biodiversity, thereby weakening the ecosystem’s self-regulation and recovery capacity (Bielen et al., 2017).

**Figure 5:**
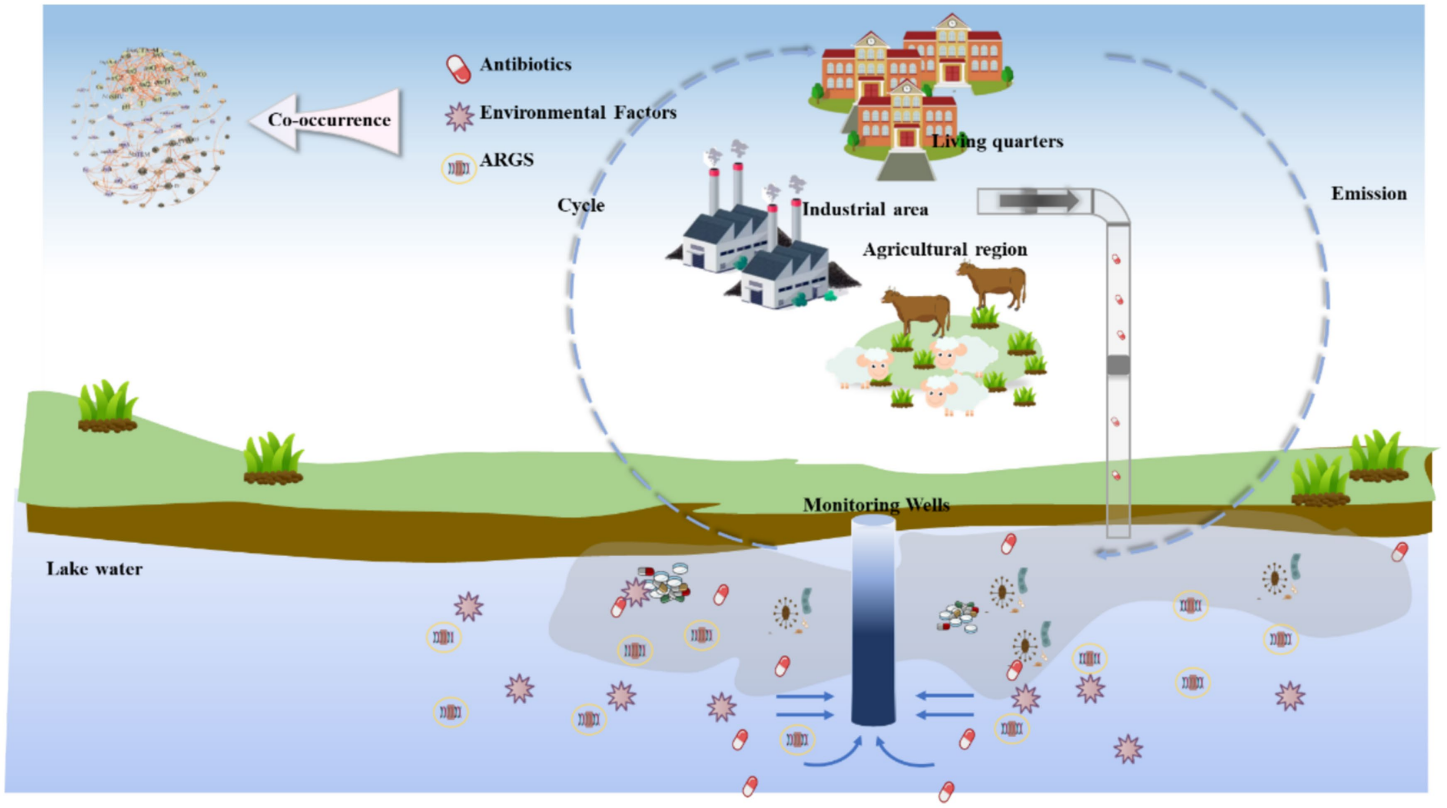
Identification of the occurrence and sources of antibiotics and antibiotic resistance genes in groundwater.

Given these concerns, the academic community widely recognizes the importance of strengthening the monitoring and risk assessment of resistance genes and their carriers in watersheds (Kraemer et al., 2019). The integrated application of molecular biology, microbiome analysis, and ecotoxicology to conduct quantitative and qualitative analyses of the types, abundance, and distribution of resistance genes is a crucial approach to assessing environmental health risks (Zhou et al., 2023). Additionally, adopting advanced wastewater treatment technologies and agricultural management practices to reduce the discharge of antibiotics and other pollutants can help mitigate the accumulation of resistance genes in the environment from the source (Figure 5).

## 7 Conclusion

Microorganisms play essential roles in the carbon and nitrogen cycles, making them indispensable for maintaining the health and functionality of watershed ecosystems. Their activities drive essential processes such as carbon sequestration, nitrogen fixation, and pollutant degradation. However, human activities have led to significant alterations in microbial communities, with potential consequences for ecosystem stability and public health. The spread of antibiotic resistance genes (ARGs) in watersheds highlights the need for better monitoring and management practices to mitigate environmental impacts. Future research should prioritize the integration of multi-omics technologies (metagenomics, metatranscriptomics, and proteomics) with advanced modeling frameworks to disentangle the complex interactions within microbial communities and their resulting functions in carbon and nitrogen cycling. This knowledge is critical for developing predictive models and effective management strategies to enhance ecosystem resilience, promote carbon sequestration, and mitigate nitrogen pollution and the spread of antibiotic resistance in watersheds facing global change.
